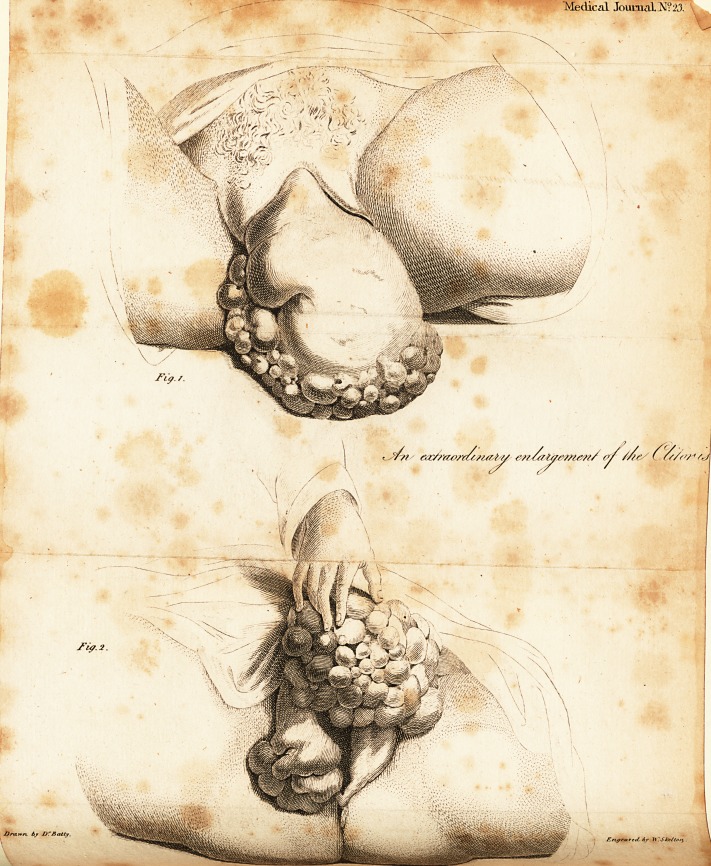# Case of an Extraordinary Enlargement of the Clitoris

**Published:** 1801-01

**Authors:** Richard Simmons

**Affiliations:** Member of the Royal College of Surgeons, Surgeon to the British Lying-in Hospital, Brownlow Street, &c. &c.


Medical and Phyſical Journal.

VOL. V.] JANUARY, 1801. [NO. XXIII.

### To Dr. BATTY.

DEAR SIR,

AS you did me the favour to make a drawing of the ſubject of the following caſe, and was preſent at the operation, I requeſt you will inſert the annexed account of it in your very uſeful Journal; and though it may not give riſe to many new ideas on our mode of treating ſuch caſes, it will ſhew to what an uncommon extent ſuch diſeaſes may arrive at without being particularly detrimental to the conſtitution, independent of the pain and diſtreſs ariſing from their weight. I am,

DEAR SIR,

Your obliged humble ſervant,

RICHARD SIMMONS.

---

*Case of an extraordinary Enlargement of the Clitoris. By* RICHARD SIMMONS, *Member of the Royal College of Surgeons, Surgeon to the British Lying-in Hospital, Brownlow Street, &c. &c.*

[ With a Plate. ]

ON the 28th of Feb. 1800, Catharine Talbot, a healthy looking woman, about thirty years of age, was placed under my care, in the Parochial Infirmary of St. Martin in the Fields, where ſhe had been admitted on account of her inability to follow her uſual occupation, which was that of working in the brick fields, and other laborious employments, from a ſwelling of great magnitude, as ſhe deſcribed it, hanging from her body; and which, upon examination, I found to be the *clitoris* enlarged to a moſt enormous ſize, gradually increaſing in bulk from its ſtem at the pubis. The circumference of the largeſt part meaſured fourteen inches, the circumference of the ſtem five inches, and the length of the tumor nine inches. Its general appearance was ſmooth and fleſhy, and its upper ſurface covered with cuticle, and not redder than the ſkin in general; round the bottom of the tumor, and all its under

*Fig. 1.*

*An extraordinary enlargement of the Clitoris*

*Fig. 2.*

*Drawn by Dr. Batty.*

*Engraved by W. Skelton.*

[illegible]

fu:f ce, it was very unequal, being made up of a clufter of fwellings of a globular form, of different fizes, from thofe of large grapes to the fmalleft; the colour of thefe were redder, fomewhat tranfparent and fhining, but not inflamed or painful to the touch. When the tumor was held up, a detached lobe from the right fide hung lower than the reft, having the fame globular appearances at its moft depending part. The nymphæ and labia on both fides, efpecially near the perineum, appeared as if taking on the fame uncommon action with the clitoris, and felt more tender; which might arife from the weight and preffure of the tumor, as they were not much enlarged.

The moft fingular circumftance was, that her general health was not at all affected, her appetite was good, and fhe menftruated regularly; nor did this enormous mafs produce any pain except from its weight, which gave her an uneafy fenfation at the fcrobiculus cordis, which was always relieved by fufpending the tumor, but which fhe was either in general too carelefs to attend to, or the neceffary fupport too inconvenient to her to apply conftantly.

The account fhe gave of it was, that the part (clitoris) began to enlarge about four years ago, and without any apparent caufe; and that it went on gradually increafing in fize for near three years, and that during the laft year it had enlarged very rapidly. On queftioning her very particularly, fhe confeffed having had a flight venereal complaint when fhe was about twenty years of age, but has not the leaft doubt of her having been perfectly cured of it, having fuffered no interruption to her health in any way till the commencement of the fwelling.

It will readily be granted that this was no common cafe; its extraordinary fize and fingular appearance neceffarily rendered it an object of curiofity, and I occafionally took feveral of my medical friends to examine it; many of whom had feen inftances of enlarged and difeafed nymphæ and clitoris, but never any thing like the prefent. I faw no chance of relieving the poor woman without an operation; and fhe being alfo well convinced of her conftantly remaining a burthen to herfelf and the world without its extirpation, fhe readily agreed to any plan I might propofe.

I determined therefore on ufing the knife, and requefted Dr. Batty, Mr. Ford, Mr. Blair, and Mr. Morris, to favour me with their attendance on the 26th of March, when the operation was performed in the following manner:—A circular incifion was made round the bafe of the fwelling near the pubis, beginning at the inferior part on the right fide, and ending on the oppofite fide, and afterwards continuing the diffection till the whole was removed. It was neceffary to take up one veffel fel on each fide; the three veffels of the clitoris were not materially enlarged, and were included in one ligature. The parts were dreffed fuperficially, and thirty drops of laudanum given her. I faw her again in the evening, and the laudanum was repeated; fhe flept pretty well during the night, and was tolerably free from pain. On the 27th, fome pain, tenfion and inflammation of the labia came on, accompanied with fever; fhe took the faline mixture during the day, and at night the opiate was repeated. She difcharged her urine freely. On the 28th, the tenfion and pain were increafed; and not having had a ftool fince the operation, fhe took a laxative medicine, which had effect. On the 29th, the dreffings were removed, having been loofened by a beginning fuppuration; fhe paffed the day rather better. On the 30th, the difcharge from the fore was confiderably increafed, and the pain and tenfion diminifhed; one of the ligatures came away. From this time the fore continued to difcharge freely for a few days, when both the other ligatures came off, the patient feeling extremely happy at the thoughts of having got rid of a difagreeable load, and with the profpect of the fore fpeedily healing; nor were her expectations difappointed, it gradually leffened in fize, and, by the 21ft of April, was completely healed, the labia, &c. affuming their natural appearance. She was difcharged the Infirmary before the end of the month.

It muft have occurred to thofe who have extenfive practice in midwifery, to have obferved children born with a peculiar conformation of the clitoris; inftances where it has appeared confiderably elongated, and covered with a large præputium, have given rife to miftakes of the fex of an infant, or have rendered it a doubt to which the child belonged. Specimens of this fort are to be feen in all anatomical collections, as well as examples of extraordinary enlargements of it in adults. And hiftories of fuch cafes are to be met with in Riolanus, Bartholine, Schurigius, and others, fome of whom believe the part endowed with a fimilar power to the penis in men. It would be of importance to difcover whether any artificial means, or excefs of venery, was capable of increafing its fize; in the cafe above related I could not difcover that at any period of its enlargement it had the power of erection, or was converted to any improper purpofe.

It appears that an enlarged clitoris is almoft endemial in fome countries, particularly Egypt and Darfur, where the excifion of it is very commonly practifed, and the operation is performed a little before the period of puberty, or at about the age of eight or nine years. This cuftom is mentioned by Strabo, and alfo by Albucafis, in his 7th chapter, who obferves,

 that that every parent knows when a child has theſe parts longer than ordinary, and cut and burn them off while girls are very young. De Graaf was alſo acquainted with this, and gives his approbation of the operation as highly neceſſary as well as decent, "Eſtque hujus partis chirurgia orientalibus tam neceſſaria quam decora." And Mr. Brown, our countryman, the celebrated traveller into Africa, tells us, that thirteen or fourteen young females underwent the operation in a houſe where he was. It was performed by a woman, and ſome of them complained much of the pain both at and after it. They were prevented from locomotion, but permitted to eat meat; the parts were waſhed every twelve hours with warm water, which profuſe ſuppuration rendered neceſſary; at the end of eight days the greater part were in a condition to walk, and liberated from their confinement; three or four of them remained under reſtraint till the thirteenth day. The reflection that naturally ariſes from this fact is, that there is no hazard in performing the operation at a very early period; and the ſucceſs attending the extirpation of the prodigious one in the caſe I have related, is a ſufficient evidence of the ſafety of an operation at a more advanced ſtage of the diſeaſe,

EXPLANATION of the PLATE.

Fig. 1. The upper ſide of the tumor. Fig. 2. The under part.

The weight of the tumor after extirpation was twenty-eight ounces.

## Figures and Tables

**Fig. 1. Fig. 2. f1:**